# Iodine deficiency hypothyroidism in children in recent years: a re-emerging issue?

**DOI:** 10.1530/EDM-24-0038

**Published:** 2024-06-26

**Authors:** Tejal Patel, Rachel Longendyke, Roopa Kanakatti Shankar, Nadia Merchant

**Affiliations:** 1 Division of Endocrinology, Children’s National Hospital, Washington, District of Columbia, USA; 2 Department of Pediatrics, George Washington School of Medicine, Washington, District of Columbia, USA; 3 Division of Pediatric Endocrinology, University of Texas Southwestern Medical Center, Dallas, Texas, USA

**Keywords:** Paediatric, Adolescent/young adult, Female, Male, Other, United States, Thyroid, Thyroid, Hypothyroidism, Paediatric endocrinology, Unique/unexpected symptoms or presentations of a disease, June, 2024

## Abstract

**Summary:**

Iodine nutrition is a growing issue within the USA due to newer trends of non-iodized salts. There are no recent reviews looking at the current state of iodine deficiency-induced hypothyroidism in children in the USA. We performed a retrospective chart review at our tertiary pediatric endocrine clinic; four met the diagnostic criteria for iodine deficiency defined by a low urine iodine level. We further characterized severity of disease, risk factors, goiter, thyroid labs and antibodies. All cases had significant goiter and were diagnosed within the last 2 years. One case had iodine deficiency due to no iodized salt intake along with concurrent diagnosis of developmental delay and multiple food allergies, while others involved the use of non-iodized salts. Two cases had iodine deficiency along with autoimmunity. It is critical to obtain a dietary history for all patients who present with goiter and/or hypothyroidism. There may be a need to consider reevaluating current preventative measures for iodine deficiency, especially for certain vulnerable populations such as children who do not consume iodized salt.

**Learning points:**

- In recent decades, iodine nutrition has become a growing concern due to changing dietary patterns and food manufacturing practices.
- A dietary history is crucial to obtain in children presenting with hypothyroidism and goiter, especially in children with restrictive diets due to behavioral concerns, developmental delays, or multiple food allergies.
- Of the 12 different types of salts commercially available, only table salt contains iodine in an appropriate amount; thus, individuals using specialty salts can develop mild to moderate iodine deficiency-related thyroid disease.

## Introduction

Iodine deficiency has been a leading cause of hypothyroidism worldwide, and nearly 35–45% of the world’s population has been deficient in iodine (1). Since iodine is a major constituent of thyroid hormone, nutritional deficiency may lead to significant thyroid dysfunction (2, 3). In children, clinical complications include pronounced goiter, developmental delay, and even cretinism – a syndrome characterized by severe irreversible mental disability (4, 5). The primary source of iodine is from an individual’s diet, mostly from iodized salt (6). About 150 µg of iodide is the required daily allowance for adolescents/adults to generate sufficient thyroid hormone (1). For children, the daily iodine requirement is 90 µg/day for those under 8 years and 120 µg/day for those older than 8 years (1). Generally, urine iodine concentrations are most often used to determine the iodine status in a region/population; however, multiple 24-h urine collections may be useful in providing reliable iodine status on an individual level (1).

In recent decades, iodine nutrition has become a growing concern due to changing dietary patterns and food manufacturing practices (1). Before the 1920s, iodine deficiency was common in Northern/Northwestern US regions and parts of Canada, referred to as the ‘goiter belt’, where 26–70% of children had apparent goiter (7). It has been nearly eliminated after fortification of food with iodized salt. Of the 12 different types of salts commercially available, only table salt is usually iodized (8). Recent data from large populations within the USA has shown a decrease in median urine iodine levels by 50% since the early 1970s and 1990s, although the population overall has remained sufficient (7, 8). Neonates and young children may be impacted to a larger degree due to the importance of thyroid hormone for brain development in early childhood years (1, 7). There are no studies looking at the current state of iodine deficiency hypothyroidism in children within the USA. Thus, we aimed to look at our institutional experience in outpatient pediatric endocrine clinic encounters for iodine deficiency hypothyroidism to gain more insight into this reemerging issue.

## Methods

**Figure 1:**
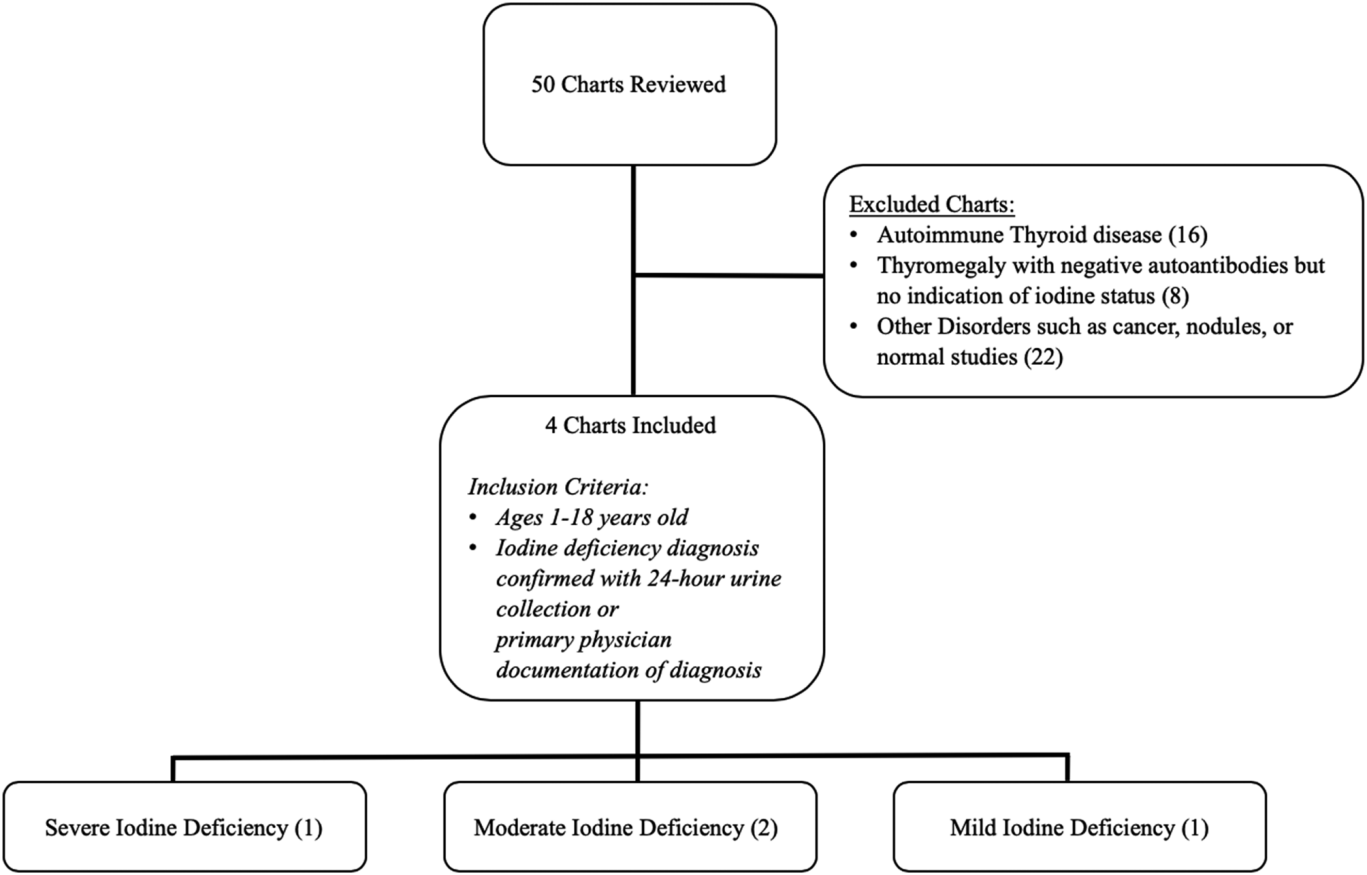
Retrospective chart review. Fifty charts were reviewed, and four met inclusion criteria.

The study was conducted retrospectively by chart review at a tertiary freestanding academic pediatric hospital outpatient endocrine clinic and was deemed exempt from institutional review board approval. Charts were identified using ICD-10 codes E01.0, E01.8 for thyromegaly, iodine-related thyroid disorder, and/or iodine deficiency hypothyroidism from January 2015 to March 2023 through a platform for electronic health records to be queried and extracted. Data was entered into a secure RedCap (Research Electronic Data Capture) database to allow for analysis of labs, goiter, diet, treatment, and confirmation of iodine deficiency (9). A 24-h urine iodine level was needed to confirm the diagnosis. Iodine reference ranges (at a population level) were used to define the level of deficiency as follows; < 20 µg/L as severe deficiency, 20–49 µg/L as moderate deficiency, 50–99 µg/L as mild deficiency, and 100–199 µg/L as adequate. Fifty charts were identified and reviewed; four met inclusion criteria for confirmed iodine deficiency by 24-h urinary iodine measurement (Fig. 1).

## Cases

All cases had significant goiter relative to thyroid labs and were diagnosed recently, 2021–2023 (Table 1).

**Table 1:** Confirmed Cases of Iodine Deficiency Hypothyroidism based on demographics, medical history, laboratory findings, and treatment. Iodine reference ranges (at population level): < 20 µg/L: severe deficiency; 20–49 µg/L: moderate deficiency; 50–99 µg/L: mild deficiency; 100–199 µg/L: adequate.

	Cases			
	1	2	3	4
Age, years	5	5	12	12
Sex	Male	Male	Female	Female
Initial labs*				
FT4, ng/dL	0.82**	1.06	0.72	0.23
TSH, mIU/mL	11.17	6.72	14.52	233
24-h iodine, μg/L	< 5	47.6	35.2	66.2
Thyroid Ab				
TPO Ab	Negative	Negative	Positive	Positive
TG Ab	Negative	Negative	Positive	Negative
Medical and nutritional history	Developmental delay, food allergies, no salt intake, no processed foods	Uses sea salt	Non iodized salt intake; recent salt loading	Kosher/Himalayan salt, cooks with iodized salt
Intervention	Levothyroxine, iodized salt, multivitamins	Iodized salt	Iodine-rich foods	Levothyroxine, iodized salt
Follow-up				
FT4, ng/dL	0.59	0.99	1.0	0.97
TSH, mIU/mL	1.67	4.7	4.29	9.49
24-h iodine, μg/L	–	64.6	132.9	–

*Reference ranges: free T4 (0.78–1.34 ng/dL); TSH (0.70–4.01 mIU/mL); **Normal free T4 for age: 1.1–1.6 ng/dL.

FT4, free T4; TSH, thyroid-stimulating hormone; TG, thyroglobulin; TPO, thyroid peroxidase antibody; Ab, antibody.

Case 1: A 5-year-old male with no known medical history presented with pronounced goiter and hypothyroidism. Parents endorsed restrictive eating behaviors, including issues with textured foods, and multiple food allergies (not formally tested), which had limited his diet to mainly pureed blends of bananas and blueberries. Initial thyroid labs showed borderline low free thyroxine and elevated TSH along with negative thyroid autoantibodies. Neck x-ray and thyroid ultrasound showed diffuse thyroid enlargement. Subsequently, a 24-h urine iodine level was obtained based on his dietary history, which was found to be < 5 µg/L, thus defined as severe iodine deficiency. He was started on levothyroxine, a multivitamin, and encouraged to increase iodized salt intake. Over time, his goiter size significantly reduced. His levothyroxine dose was titrated based on thyroid hormone levels and he was ultimately taken off after 20 months of treatment. In the interim, he was diagnosed with G6PD deficiency, developmental delay concerning probable autism, and eosinophilic esophagitis.

Case 2: A 5-year-old male presented with subclinical hypothyroidism. He had abnormal thyroid labs and poor growth and was found to have thyromegaly on examination. Dietary history was notable for sea salt use at home. Laboratory workup showed a normal free thyroxine level with an elevated TSH and negative thyroid antibodies. His 24-hour urine iodine level was initially measured to be 47.6 µg/L, considered moderate iodine deficiency. There was improvement in thyroid size as well as normalization in thyroid hormone levels after the incorporation of iodized salt in his diet. His 24-h urine iodine level improved from 47.6 to 64.6 µg/L in a time span of 6 months with normalization of thyroid labs.

Case 3: A 12-year-old female with positive thyroid antibodies was found to also have moderate iodine deficiency hypothyroidism; her initial 24-h urine iodine level was 35.2 µg/L. She presented with abnormal thyroid labs, neck swelling, and a low urine iodine level, which were performed by her pediatrician. Her dietary history was notable for the AIP (Autoimmune Protocol) diet, (no legumes, no gluten, no dairy, no seeds, no grains, and no nightshades). She had started ‘salt loading’ as per recommendations from a naturopathic doctor. Laboratory workup showed low free thyroxine and elevated TSH, along with two positive thyroid antibodies (thyroid peroxidase (TPO) and thyroglobulin). Levothyroxine and iodized salt were recommended; however, the family did not start levothyroxine treatment. She had normalization of urine iodine level, thyroid hormone, and TSH level after kelp supplementation and increased fish intake in her diet, thus had remained off levothyroxine.

Case 4: A 12-year-old female with mild iodine deficiency hypothyroidism with one positive thyroid antibody. At presentation, she had thyromegaly (one side greater than the other) with labs concerning severe hypothyroidism. She was found to have one positive thyroid antibody (thyroid peroxidase antibody, TPO). Thyroid ultrasound showed a diffusely enlarged gland. She was started on levothyroxine but did not have normalization of thyroid levels along with no reduction in her goiter size. Dietary history was notable for kosher and Himalayan salt use with iodized salt restricted to baking products. Her 24-h urine iodine level was initially 66.2 µg/L. She remained on levothyroxine, and her thyroid levels normalized along with a reduction in her goiter size until dietary changes were made.

## Discussion

Iodine deficiency may not be routinely considered as a cause of hypothyroidism by clinicians in developed countries. A few cases of iodine deficiency secondary to a restrictive diet in children with food allergies or behavior concerns have been reported in the literature (10). Case 1 was similar, with dietary limitations and developmental delay, who presented with significant goiter despite negative anti-thyroid antibodies. His diagnosis was made by exploring his nutritional history and confirming a low urine iodine level after confirming negative thyroid antibodies. While iodine deficiency is the leading cause of hypothyroidism worldwide, autoimmune thyroiditis is the most common cause of hypothyroidism in developed countries such as the USA. Therefore, most clinicians do not routinely explore other etiologies of hypothyroidism, such as iodine deficiency, unless anti-thyroid antibodies are not detected. Based on our review, a history of behavioral concerns with dietary restrictions should prompt evaluation for sufficiency of iodine intake from the start.

The shift in dietary practices involving the use of specialty (non-iodized) salts increases the risk of developing iodine deficiency hypothyroidism, which may present in milder forms. In our cohort, three children were evaluated for goiter and had mild to moderate iodine deficiency due to the intake of specialty salts and a limited processed food diet. Two children with abnormal thyroid function and positive thyroid antibodies were also identified as being iodine-deficient due to non-iodized salt use. This supports the importance of inquiring about the type of salt being consumed to ensure that iodine deficiency is not a contributory factor, especially in those with significant goiter with hypothyroidism, even with positive thyroid antibodies. Moreover, two of these cases with mild-to-moderate deficiency, one of whom also had autoimmunity, showed improvement in thyroid labs by simply adding iodized salt or iodine-containing foods without requiring any levothyroxine. This reinforces the importance of taking a dietary history since actions to increase iodine intake can reverse the thyroid disease over time.

The limitation of this retrospective case review includes a small sample size. Cases were initially identified based on ICD-10 codes. While fifty cases were identified, this method of screening may not have identified all relevant cases based on how they were coded. Another major limitation includes using 24-h urine iodine levels to confirm the diagnosis. Multiple 24-h urine collections are ideal to determine the iodine status of an individual, but it can be difficult to obtain in a pediatric patient, therefore making the diagnosis of iodine deficiency more challenging. For this reason, it is often difficult to confirm the diagnosis. While our review only identified four cases, it is likely that there have been more individuals with hypothyroidism actually due to unconfirmed iodine deficiency. A spot urine collection may be more feasible in this population; however, it is quantified in relation to urinary creatinine excretion and does not appear to be a reliable screening method, especially since it would provide iodine status around the time the sample is drawn, which varies throughout the day. A 24-h urine collection (measured as UI) is a more accurate and reliable method to measure urinary iodine excretion and thereby iodine intake.

While generalizations on a population level cannot easily be deduced from our cases, they highlight the importance of dietary history-taking in vulnerable populations such as children to assess iodine intake and its role in thyroid function. Also, current trends need to be assessed nationally and globally to determine if preventative health measures need to be reevaluated.

## Declaration of interest

TP received a grant from Dexcom, Inc. to get supplies for research pertaining to a type 2 diabetes study; there is no conflict of interest related to the current study topic. NM was on the advisory board of BioMarin, Pfizer, and Alexion; there is no conflict of interest in relation to the current paper or topic. RKS received an investigator-initiated research grant from BioMarin to fund a study of vosoritide in girls with Turner syndrome; there is no conflict of interest related to the current study topic. The other authors have no conflicts of interest to disclose.

## Funding

This study did not receive any specific grant from any funding agency in the public, commercial, or not-for-profit sector.

## Patient consent

This study protocol was reviewed and approved by Children’s National Hospital Institutional Review Board (IRB) (approval no. STUDY00000555). The study has been granted an exemption from requiring written informed consent by the IRB. Thus, consent was not obtained. The data collected for this retrospective chart review are available in REDCap format.

## Author contribution statement

Patel conceptualized and designed the study, designed the data collection instruments, collected data, carried out the initial analyses, drafted the initial manuscript, and revised the manuscript. Longendyke and Shankar collected data, carried out the initial analyses, critically reviewed, and revised the manuscript. Merchant conceptualized the study, collected data, carried out the initial analyses, critically reviewed, and revised the manuscript. All authors approved the final version of the manuscript and agreed to be accountable for all aspects of the work.

## References

[1] Hatch-McChesney A & Lieberman HR. Iodine and iodine deficiency: a comprehensive review of a re-emerging issue. Nutrients 2022 14 3474. (10.3390/nu14173474) PMC9459956 36079737

[2] Babiker A Alawi A Al Atawi M & Al Alwan I. The role of micronutrients in thyroid dysfunction. Sudanese Journal of Paediatrics 2020 20 13–19. (10.24911/SJP.106-1587138942) 32528196 PMC7282437

[3] Stone MB Wallace RB & Institute of Medicin e (U.S.). Committee on Medicare Coverage of Routine Thyroid Screening. In Medicare Coverage of Routine Screening for Thyroid Dysfunction. National Academies Press, 2003. (10.17226/10682) 25057658

[4] Delange F. The disorders induced by iodine deficiency. Thyroid 1994 4 107–128. (10.1089/thy.1994.4.107) 8054857

[5] Angermayr L & Clar C. Iodine supplementation for preventing iodine deficiency disorders in children. Cochrane Database of Systematic Reviews 2004 2 CD003819. (10.1002/14651858.CD003819.pub2) 15106221

[6] Krela-Kaźmierczak I Czarnywojtek A Skoracka K Rychter AM Ratajczak AE Szymczak-Tomczak A Ruchała M & Dobrowolska A. Is there an ideal diet to protect against iodine deficiency? Nutrients 2021 13 513. (10.3390/nu13020513) 33557336 PMC7914421

[7] Leung AM Braverman LE & Pearce EN. History of U.S. iodine fortification and supplementation. Nutrients 2012 4 1740–1746. (10.3390/nu4111740) 23201844 PMC3509517

[8] What is iodine and what does it do? How much iodine do I need? Available at: http://ods.od.nih.gov.

[9] Harris PA Taylor R Thielke R Payne J Gonzalez N & Conde JG. Research Electronic Data Capture (REDCap)--a metadata-driven methodology and workflow process for providing translational research informatics support. Journal of Biomedical Informatics 2009 42 377–381. (10.1016/j.jbi.2008.08.010) 18929686 PMC2700030

[10] Booms S Hill E Kulhanek L Vredeveld J & Gregg B. Iodine deficiency and hypothyroidism from voluntary diet restrictions in the US: case reports. Pediatrics 2016 137 e20154003. (10.1542/peds.2015-4003) 27244854

